# Hippocampal neural circuit connectivity alterations in an Alzheimer’s disease mouse model revealed by monosynaptic rabies virus tracing

**DOI:** 10.1016/j.nbd.2022.105820

**Published:** 2022-07-14

**Authors:** Qiao Ye, Gocylen Gast, Xilin Su, Takashi Saito, Takaomi C. Saido, Todd C. Holmes, Xiangmin Xu

**Affiliations:** a Department of Anatomy and Neurobiology, School of Medicine, University of California, Irvine, CA 92697, USA; b Department of Biomedical Engineering, University of California, Irvine, CA 92697, USA; c Department of Physiology and Biophysics, School of Medicine, University of California, Irvine, CA 92697, USA; d Center for Neural Circuit Mapping, University of California, Irvine, CA 92697, USA; e Department of Neurocognitive Science, Institute of Brain Science, Nagoya City University Graduate School of Medical Sciences, Nagoya, Aichi 467-8601, Japan; f Lab for Proteolytic Neuroscience, RIKEN Center for Brain Science, Wako, Saitama 351-0106, Japan

**Keywords:** Alzheimer’s disease, hippocampus, CA1, Neural circuit, Retrograde, Rabies tracing, Monosynaptic

## Abstract

Alzheimer’s disease (AD) is a progressive neurodegenerative disorder with growing major health impacts, particularly in countries with aging populations. The examination of neural circuit mechanisms in AD mouse models is a recent focus for identifying new AD treatment strategies. We hypothesize that age-progressive changes of both long-range and local hippocampal neural circuit connectivity occur in AD. Recent advancements in viral-genetic technologies provide new opportunities for semi-quantitative mapping of cell-type-specific neural circuit connections in AD mouse models. We applied a recently developed monosynaptic rabies tracing method to hippocampal neural circuit mapping studies in AD model mice to determine how local and global circuit connectivity to hippocampal CA1 excitatory neurons may be altered in the single *APP* knock-in (APP-KI) AD mouse model. To determine age-related AD progression, we measured circuit connectivity in age-matched littermate control and AD model mice at two different ages (3–4 vs. 10–11 months old). We quantitatively mapped the connectivity strengths of neural circuit inputs to hippocampal CA1 excitatory neurons from brain regions including hippocampal subregions, medial septum, subiculum and entorhinal cortex, comparing different age groups and genotypes. We focused on hippocampal CA1 because of its clear relationship with learning and memory and that the hippocampal formation shows clear neuropathological changes in human AD. Our results reveal alterations in circuit connectivity of hippocampal CA1 in AD model mice. Overall, we find weaker extrinsic CA1 input connectivity strengths in AD model mice compared with control mice, including sex differences of reduced subiculum to CA1 inputs in aged female AD mice compared with aged male AD mice. Unexpectedly, we find a connectivity pattern shift with an increased proportion of inputs from the CA3 region to CA1 excitatory neurons when comparing young and old AD model mice, as well as old wild-type mice and old AD model mice. These unexpected shifts in CA3-CA1 input proportions in this AD mouse model suggest the possibility that compensatory circuit increases may occur in response to connectivity losses in other parts of the hippocampal circuits. We expect that this work provides new insights into the neural circuit mechanisms of AD pathogenesis.

## 1. Introduction

Dementia and age-related cognitive decline are major health concerns worldwide. Alzheimer’s disease (AD) is the most common form of age-progressive adult dementia. AD is characterized by distinguishable pathological features that include synaptic and neuronal loss, and age-progressive deterioration of cognitive function. In keeping with the original clinical observations of Alois Alzheimer, the longstanding focus in AD research has been the pathological accumulation of amyloid-beta (Aβ) protein fragments (Querfurth and LaFerla, 2010; Selkoe, and American College of, P., and American Physiological, S, 2004) and hyperphosphorylated tau proteins (Kosik et al., 1986). Aβ results from aberrant cleavage of the amyloid precursor protein (APP) by β- and γ-secretases. Excessive Aβ production causes fibril aggregation that culminates in AD neuropathology (Hardy and Allsop, 1991; Hardy and Selkoe, 2002; Soria Lopez et al., 2019).

While the mechanistic basis of AD pathogenesis has been studied for many years, more recent evidence suggests that AD can be functionally characterized by the disruption of both long-range and local neural circuit connections (Busche et al., 2015; Geula, 1998; Harris et al., 2020; Jeon et al., 2018; Neuman et al., 2015; Palop et al., 2007; Palop and Mucke, 2010). Dil-based retrograde neural circuit tracing of the hippocampus (via bulk injection targeted in the dentate gyrus) in the 5xFAD mouse model qualitatively suggests decreased hippocampal connectivity between the hippocampus and the medial septum and diagonal band (MS-DB), entorhinal cortex (EC), auditory cortex, locus coeruleus, dorsal raphe, substantia nigra pars compacta, and olfactory bulb (Jeon et al., 2018). Hippocampal CA1, a brain region associated with spatial learning and memory, is one of the most vulnerable brain regions in AD (Andrade-Moraes et al., 2013; Hyman et al., 1990; Montero-Crespo et al., 2021; West et al., 2004). *In vivo* electrophysiological recordings in a single *APP* knock-in (APP-KI) AD mouse model reveals that hippocampal CA1 place cells exhibit spatial memory defects, including disrupted spatial remapping, due to deteriorated circuit connections between CA1 and entorhinal cortex (EC) in APP-KI mice (Jun et al., 2020). Miniscope-based calcium imaging of CA1 neurons in freely behaving triple-transgenic (3xTg) AD model mice shows spatial encoding defects in CA1 circuit ensemble activities even preceding AD pathology and AD-related memory behavioral deficits (Lin et al., 2022).

In this study using monosynaptic rabies virus tracing, we measured local and global circuit connectivity to hippocampal CA1 excitatory neurons to test our hypothesis that these connections are altered in the single APP^NL-G-F^ KI AD mouse model (Saito et al., 2014). Recent advancements in viral-genetic-based retrograde tracing provide new opportunities for semi-quantitative mapping of cell-type-specific neural circuit connections (Sun et al., 2019; Sun et al., 2014; Wickersham et al., 2007; Xu et al., 2016; Xu et al., 2020). The genetically modified monosynaptic rabies virus tracing technique is useful for mapping the direct circuit inputs to specific types of neurons. We take advantage of the ability to target rabies infection to specific cell types using EnvA pseudotyping, and to limit trans-synaptic spread to direct inputs, by using glycoprotein gene-deleted (ΔG) rabies virus and transcomplementation. Specifically, ΔG rabies virus (deletion mutant) is pseudotyped with the avian sarcoma leucosis virus glycoprotein EnvA (EnvA RVΔG), which can only infect neurons that express avian tumor virus receptor A (TVA), an avian receptor protein that is absent in mammalian cells unless it is provided through secondary exogenous gene delivery. We use helper AAV to provide both TVA and rabies glycoprotein expression in CA1 excitatory cells. The deletion-mutant rabies virus (RVΔG) can then be transcomplemented with the rabies glycoprotein in the same TVA-expressing cells to enable its retrograde spread restricted to direct presynaptic neurons.

The mouse model used in this study is the APP^NL-G-F^ KI mouse line (Saito et al., 2014), with the knocked-in human *APP* gene with multiple mutations: Swedish (KM670/671NL), Beyreuther/Iberian (I716F) and Arctic mutations (E693G). There are interpretational advantages of using the knock-in approach: pathogenic Aβ levels are elevated in the APP^NL-G-F^ KI mouse. Furthermore, potential APP overexpression artifacts are not an interpretational issue with this AD model mouse line. To examine age-related progression using APP^NL-G-F^ KI versus age-matched control mice, we studied littermate control wild-type mice that share the same genetic background and AD model mice at two different ages (3–4 vs. 10–11 months old). These ages were chosen based on previously established findings of behavioral performance defects and neuropathological features (Mehla et al., 2019; Saito et al., 2014). Our results show alterations in circuit connectivity of hippocampal CA1 in AD model mice, with overall weaker extrinsic connectivity strengths,connectivity pattern shifts in AD model mice compared with control mice.

## 2. Materials and methods

### 2.1. Animals

All experiments were conducted according to the National Institutes of Health guidelines for animal care and use, and were approved by the University of California, Irvine Institutional Animal Care and Use Committee (IACUC, protocol #: AUP-20-002) and Institutional Biosafety Committee (IBC). To study the extrinsic CA1 excitatory circuit connections, the following two strains of mice were used: wild type (WT) C57BL/6 J, the young group at age 3–4 months (*n* = 7; 3 males, 4 females) and the old group at 8–11 months (*n* = 10; 6 males, 4 females), and APP-knock in (APP-KI, strain APP^NL-G-F^), the young group at 3–4 months (n = 10; 5 males, 5 females) and the old group at 8–11 months (*n* = 8; 5 males, 3 females). The mice had free access to food and water in their home cages with lights maintained on a 12-h light/dark cycle. All personnel working with the rabies virus received rabies vaccinations and experiments were conducted under biosafety level (BSL) 2 conditions.

### 2.2. Viral injections

Mice were first anesthetized under 1–2% isoflurane for 10 min with a 0.8 l/min oxygen flow rate using an isoflurane tabletop unit (HME109, Highland Medical Equipment, Temecula, CA, USA). Their head fur was shaved, then the mice were placed in a rodent stereotaxic frame (Leica Angle Two™ for mouse, Leica Biosystems Inc., Buffalo Grove, IL, USA) and continuously anesthetized with isoflurane flow at 1%. After disinfection, a small incision was made in the skin, and the skull was exposed to show the landmarks of bregma and lambda. Guided by a digital atlas of the stereotaxic machine, a three-axis micromanipulator was used to locate the desired injection site relative to bregma and lambda. A small craniotomy was made above the injection site, exposing the dura.

A glass pipette (tip inner diameter, 20–30 μm) was loaded with virus solution, lowered to the target injection site, and the virus was delivered through picospritzer (Parker Hannifin, Hollis, NH, USA) pressure injection at the rate of 20–30 nl/min with a 10 ms pulse duration. After injection, the glass pipette remained at the injection site for 10 min and was then withdrawn at a constant slow speed to prevent the backflow of the virus. After the mice were removed from the stereotaxic frame, their skin was sutured using tissue adhesive (3 M Vetbond, St. Paul, MN, USA). The mice were injected with 5 mg/kg Carprofen to mitigate pain and inflammation. The mice were put on a heating pad for 15 min and then back in their home cages. The virus was injected into the pyramidal layer of dorsal hippocampal CA1 using the following coordinates: anteroposterior (AP) −1.94 mm, mediolateral (ML) −1.40 mm, dorsoventral (DV) −1.35 mm, all values given relative to bregma.

To map the input circuit connectivity of CA1, we used the following AAVs: AAV8-DIO-TC66T-2A-GFP-2A-oG (Salk Institute, CA, US, 5.06 × 10^13^ GC/ml) and AAV1-CaMKII-HI-EGFP-Cre-WPRE-SV40 (Addgene #105551, 1.76 × 10^13^ GC/ml). The AAV8-DIO-TC66T-2A-GFP-2A-oG was 1:4 diluted with phosphate-buffered saline (PBS). The diluted AAV was 1:1 mixed with AAV1-CaMKII-HI-EGFP-Cre-WPRE-SV40. This mixture was then further diluted at a 1:4 ratio with PBS. Finally, 0.1 μl of the diluted AAV mixture was injected into the hippocampal CA1 target region on day 1. After 17 days, the mice were injected with the rabies virus EnvA-SADΔG-RV-DsRed (lab-made, 2.1 × 10^9^ IU/ml, 0.4 μl) at the same injection site using pressure injection. Rabies virus was made locally at the Center for Neural Circuit Mapping Center (CNCM) of the University of California, Irvine, with required cell lines and seeding viruses originally from Dr. Edward Callaway’s group at the Salk Institute for Biological Studies. The rabies virus was allowed to replicate and retrogradely spread from targeted Cre + cell types to directly connected presynaptic cells for 9 days before the mice were perfused for tissue processing.

### 2.3. Histology and immunochemistry

The mice were perfused with 20 ml of PBS, followed by 40 ml PBS containing 4% paraformaldehyde (PFA) using a mini-pump with variable flow (United States Plastic Corp., US). The perfused brains were fixed in 4% PFA solution for 24 h and then soaked in a 30% sucrose-PBS solution for another 24 h at 4°C. Then the brains were frozen in dry ice and coronally sectioned at 30 μm thickness using a microtome (Leica SM2010R, Germany). One out of every three consecutive sections were mounted, coverslipped, and imaged. To examine the expression of amyloid-beta, two sections from each of five brains from each mouse group (10 sections in total for each mouse group) were stained with primary antibody 6E10 (Biolegend, Mouse, #803002,1:500 dilution, US), followed by Cy3 conjugated donkey anti-mouse secondary antibody (Jackson ImmunoResearch, 1:200 dilution, US). All sections were counterstained with 10 μM DAPI. Images were acquired by an automated fluorescent slide scanner (Olympus VS120-S6 slide scanner, Japan) using a 10× magnification objective. For higher resolution imaging, selected slices were imaged using a confocal microscope (Olympus FLUOVIEW FV3000, Japan) with a 40× magnification objective.

### 2.4. Data quantification and statistics

The amyloid plaque number and size and the CA1 area or field of view (FOV), were quantified unbiasedly using the 2-point circle measurement tool in Olympus VS-ASW software (Olympus, Japan). The plaque intensity was measured using the mean gray value measurement tool in Fiji ImageJ. Two samples from each mouse brain were quantified. The two measurements were averaged for each brain for statistical comparisons.

For rabies tracing experiments, we followed the established counting protocol in the previous publication (Sun et al., 2014). We first selected the brain section with the target region, CA1 to identify EGFP and DsRed doubled-labeled starter neurons that are restricted to CA1. All the starter cells were manually counted using the counting tool in Adobe Photoshop (Adobe, San Jose, CA, US). Next, we aligned the rest of the viral-infected brain sections with a standard Allen mouse brain atlas (http://atlas.brain-map.org/atlas) to determine the anatomical structures for the quantification of labeled cells in specified brain regions. No stereological measurement protocol was used; all labeled cells in each section of the brain section series (i.e., 1 out of every 3 sections was mounted for examination of virally labeled neurons in different brain structures) were counted. We operationally defined the input connectivity strength index (CSI) as the ratio of the number of presynaptic neurons in a brain region versus the number of starter neurons in the CA1 region. The CSI values allow us to quantitatively compare input strengths from different brain regions to CA1 excitatory neurons. We calculated the proportion of input (PI) index as the ratio of the number of labeled presynaptic neurons in a brain region of interest versus the overall total labeled neurons in each case.

The anterior to posterior (AP) CSI distribution curve was plotted to show the input pattern across the AP axis within mouse brains. For each mouse, an input value is determined for every brain slice, which corresponds to an AP number (relative to bregma) according to the standard Allen mouse brain atlas (http://atlas.brain-map.org/atlas). For all mice within the same age/genotype group, the CSI values were registered to similar AP numbers.

All data are presented as the mean ± SE. We applied appropriate statistical tests, and the data analysis was conducted using GraphPad Prism (GraphPad Software, San Diego, CA, USA) or MATLAB scripts. Statistical analysis methods included the Wilcoxon rank-sum test, paired Wilcoxon rank sum test, and linear mixed effects model (LME) (Yu et al., 2022). Alpha levels of *p* ≤ 0.05 were considered significant. Different levels of statistical significances are represented by * *p* < 0.05, ** *p* < 0.01, *** *p* < 0.001, **** *p* < 0.0001.

## 3. Results

### 3.1. Age-progressive amyloid-beta deposition in CA1 of APP-KI mice

**Fig. 1.**
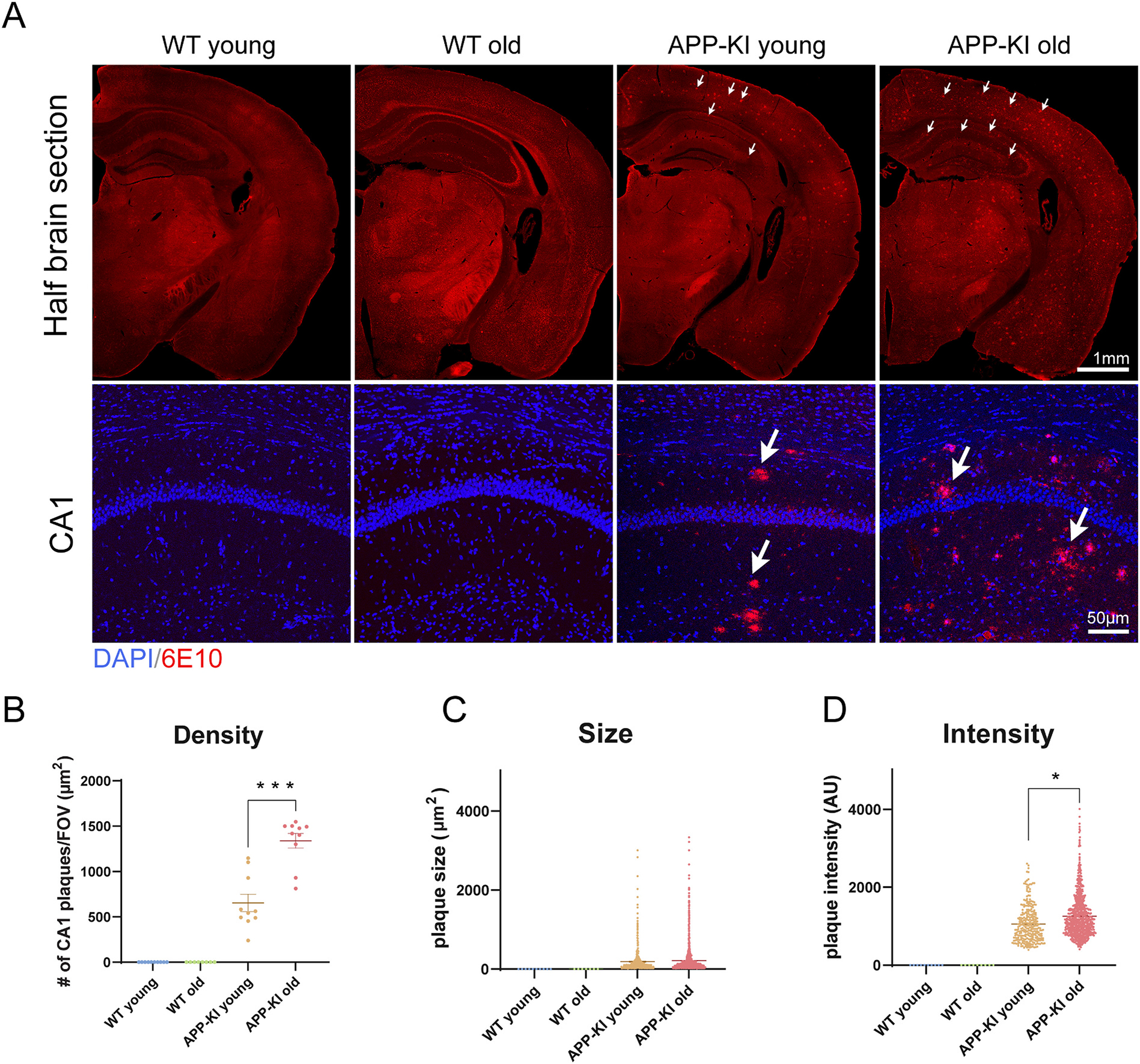
Age-progressive Aβ pathology in APP-KI mice. A) Representative images of amyloid plaques in age-matched young and old APP-KI and WT mice. The upper four panels show half coronal brain sections in red channel autofluorescence without immunostaining. The bottom four panels depict enlarged views of 6E10 amyloid antibody immunostained hippocampal CA1 slices. White arrows point to the amyloid plaques in brain slices prepared from APP-KI young and APP-KI old mice. No plaques are detected in WT young and old mice. More amyloid deposits are detected in APP-KI old mice relative to APP-KI young mice. DAPI is labeled blue, amyloid plaque is labeled red. Scale bars are in figure panels. B–D) Quantification of amyloid plaques stained by 6E10 antibody. No amyloid plaques are found in either WT young or old mice, but in APP-KI mice there is a significant age-dependent increase in both plaque density and intensity (B, density, APP-KI young: 654.3 ± 94.9, APP-KI old: 1339 ± 81.3, Wilcoxon rank-sum test, *p* = 2.0 × 10^−4^; D, intensity, APP-KI young: 1057 ± 27.4, APP-KI old: 1254 ± 18.9, linear mixed effects model, *p* = 0.045573). C, no significance is detected in plaque size measurement. Two brain sections from each of 5 brains from each group of mice were used for quantification. AU: arbitrary unit. * *p* < 0.05, ** *p* < 0.01, *** *p* < 0.001. (For interpretation of the references to colour in this figure legend, the reader is referred to the web version of this article.)

To detect the level of Aβ plaques in age-matched APP-KI and WT mice, we stained coronal brain sections with the 6E10 Aβ monoclonal antibody (Kim et al., 1988) and performed plaque quantification (Fig. 1). Two age groups of mice were selected, at 3–4 months old (young group), in which early plaque formation can be detected in APP^NL-G-F^ KI mice, and at 10–11 months (old group), for which cognitive defects are observed in APP^NL-G-F^ KI mice at this age (Saito et al., 2014). Half brain sections with autofluorescence in the red channel reveal distinct plaque patterns in APP-KI young and old mice hippocampus and cortex, even in the absence of immunostaining (Fig. 1A). Aβ antibody 6E10 immunostained CA1 images show extensive amyloid plaques in APP-KI mice at greater resolution (Fig. 1A). There are significantly more 6E10-stained plaques in APP-KI old brains relative to APP-KI young brains, consistent with the age-progressive nature of AD. In contrast, no 6E10-immunopositive plaques are detected in WT young and old mice. Quantitative plaque analysis of 6E10 staining shows that APP-KI old mice have both significantly higher plaque density and stronger plaque immunofluorescence intensity in hippocampal CA1 relative to young APP-KI mice (Fig. 1B and D; density, APP-KI young: 654.3 ± 94.9, APP-KI old: 1339 ± 81.3, Wilcoxon rank-sum test, *p* = 2.0 × 10^−4^; intensity, APP-KI young: 1057 ± 27.4, APP-KI old: 1254 ± 18.9, linear mixed effects model, *p* = 0.045573). No significant differences in plaque size are detected between the two ages of APP-KI mice (Fig. 1C). These results show age-progressive Aβ pathology development in APP-KI mice, consistent with the earlier reports (Saito et al., 2014).

### 3.2. Monosynaptic rabies tracing in age-matched APP-KI and control mice and overall compromised brain-wide connectivity in APP-KI mice

**Fig. 2.**
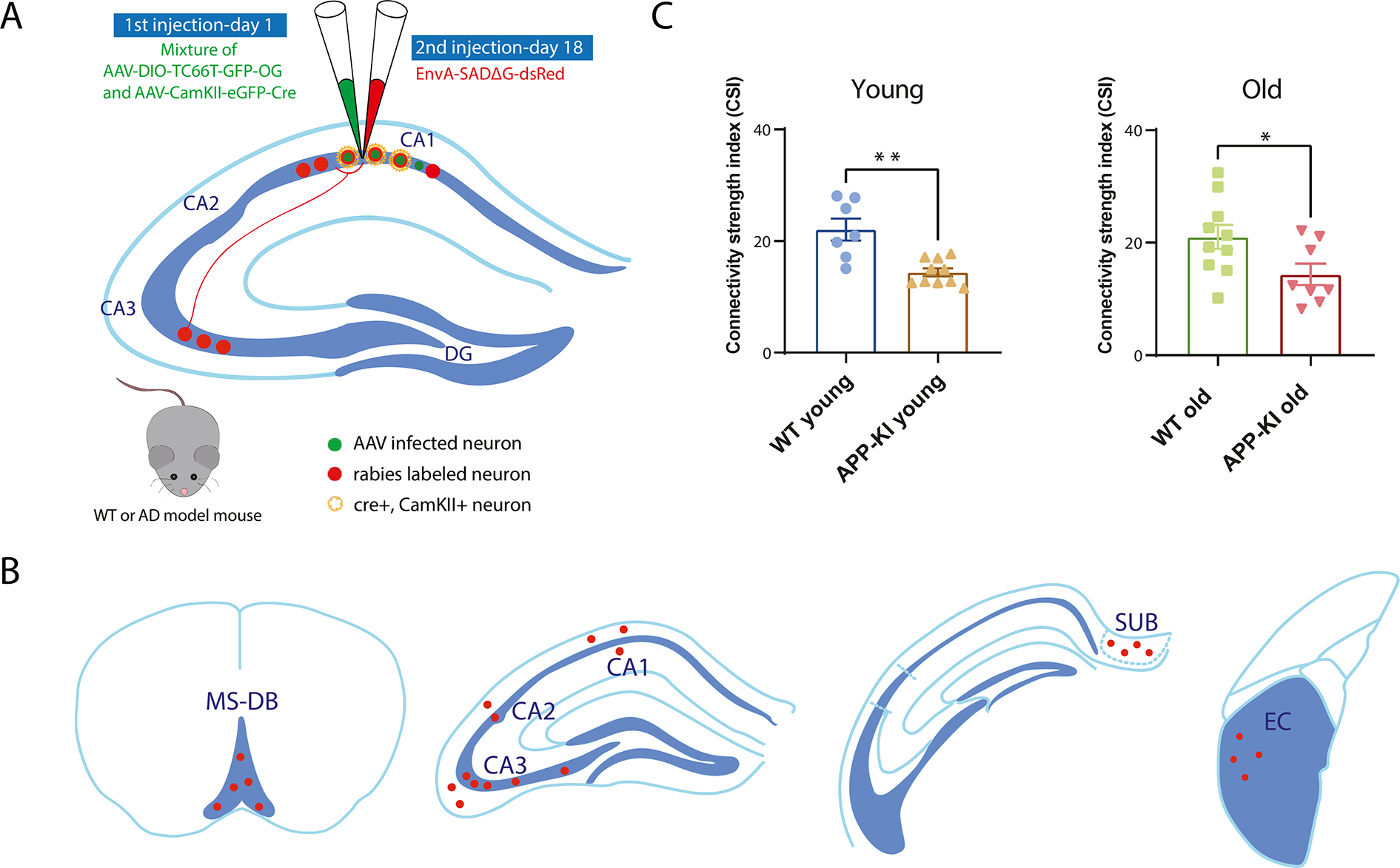
Cre-dependent monosynaptic rabies tracing reveals overall reduced circuit connectivity in APP-KI mice. A) Schematic of cell-type-specific retrograde monosynaptic rabies tracing. To specifically label excitatory CA1 neurons, AAV helper virus (AAV-DIO-TC66T-GFP-oG and AAV-CaMKII-EGFP-Cre), labeled green, was injected into the dorsal hippocampal CA1 pyramidal layer, followed 17 days later at the same site by an injection of rabies virus (EnvA-SADG-DsRed), labeled red. The neurons labeled both green and red represent the starter neurons. The neurons labeled only red represent the presynaptic inputs to the starter neurons. B) Major input regions to CA1 pyramidal layer excitatory neurons, including MS-DB, CA1, CA2, CA3, SUB, and EC. Red colour labels represent presynaptic input neurons. C) Overall connectivity revealed by monosynaptic rabies tracing. Left to right, wild type and APP-KI mice at young and old age. The connectivity strength is determined by the overall number of labeled neurons in the whole brain divided by the total number of starter neurons. APP-KI young mice (*n* = 10) have significantly weaker connectivity strength compared to WT young mice (*n* = 7) (WT young: 22.10 ± 1.97, APP-KI young: 14.41 ± 0.72, Wilcoxon rank-sum test, p = 2.0 × 10^−3^). APP-KI old mice (*n* = 8) have significantly weaker connectivity strength relative to WT old mice (n = 10) (WT old: 21.01 ± 2.14, APP-KI old: 14.36 ± 1.92, Wilcoxon rank-sum test, *p* = 3.40 × 10^−2^). (For interpretation of the references to colour in this figure legend, the reader is referred to the web version of this article.)

**Fig. 3.**
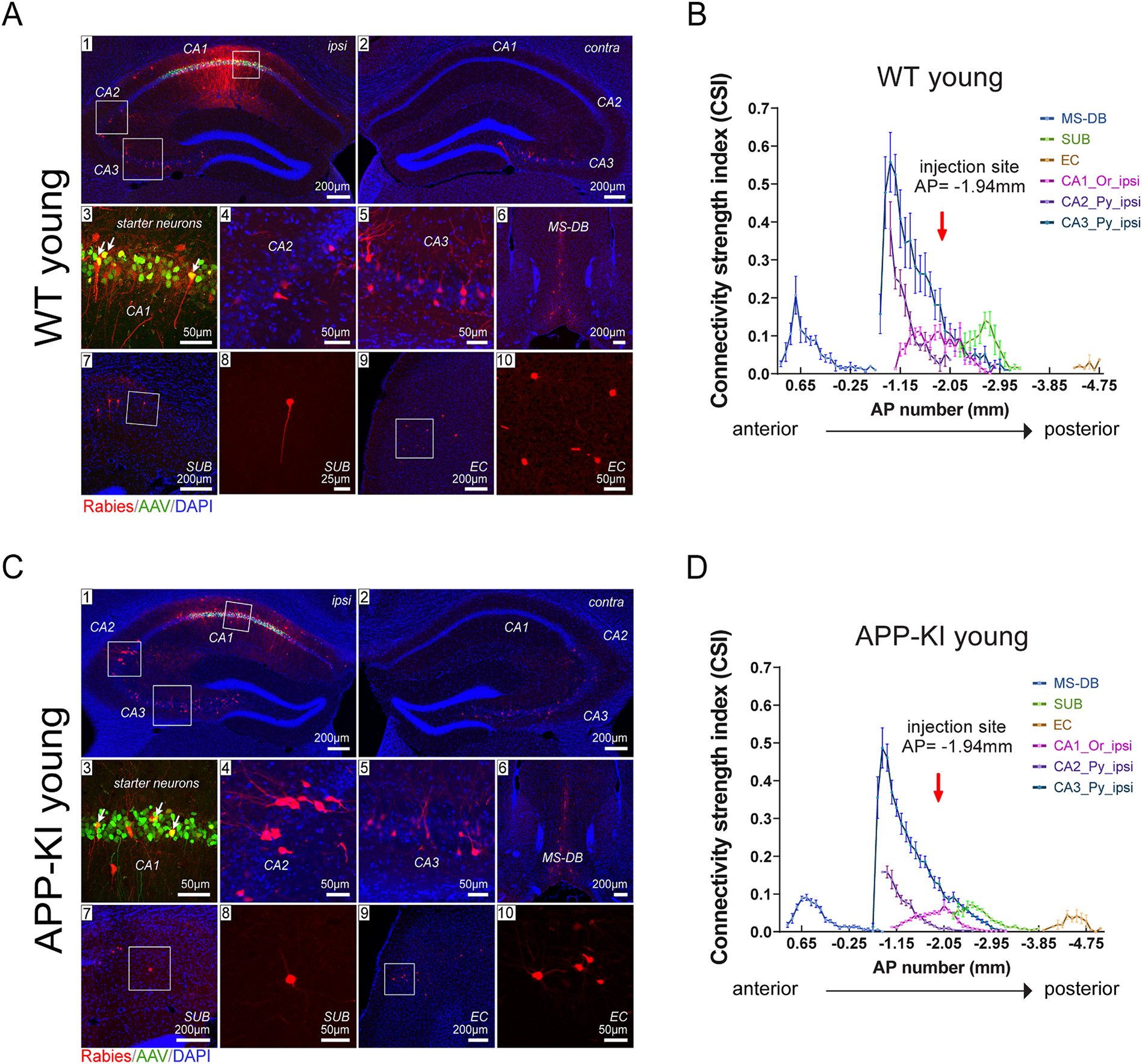
Monosynaptic rabies tracing maps specific regional inputs to excitatory CA1 cells along the anterior-posterior (AP) axis in young WT and APP-KI mice. A, C) Representative fluorescent coronal section images from WT young (n = 7) and APP-KI young (n = 10), respectively. Rabies virus-infected neurons are labeled by DsRed, and AAV-infected neurons are in green. All slices are counterstained by DAPI in blue. For both A and C, (1) shows the ipsilateral (ipsi) hippocampal formation including the CA1 injection site. (2), rabies virus mapped presynaptic inputs in the contralateral (contra) hippocampus. (3), enlarged image of CA1 starter neurons expressing both EGFP and DsRed fluorescent proteins from AAV and rabies virus. For both A and C (4–10), results of rabies virus-mediated retrograde monosynaptic tracing from CA1. The input regions include hippocampal CA2 (4), CA3 (5), medial septum and diagonal band (MS-DB) (6), subiculum (SUB) (7 and 8), and entorhinal cortex (EC) (9 and 10). (3), (4), (5), (8), and (10) are enlarged views of the white boxed areas shown in (1), (7) and (9), respectively. Scale bars are labeled for each panel. B, D) The connectivity strength index (CSI) distribution along AP positions across the whole brain. The CSI is defined as the number of input neurons normalized by the number of starter neurons; the AP position is given relative to bregma values. The red arrow at AP = −1.94 mm shows the position of the injection site. Representative input regions are used for the AP plot, including the ipsilateral hippocampal CA1 oriens layer (Or), ipsilateral CA2 and ipsilateral CA3 pyramidal layers (Py), as well as MS-DB, SUB, and EC. (For interpretation of the references to colour in this figure legend, the reader is referred to the web version of this article.)

**Fig. 4.**
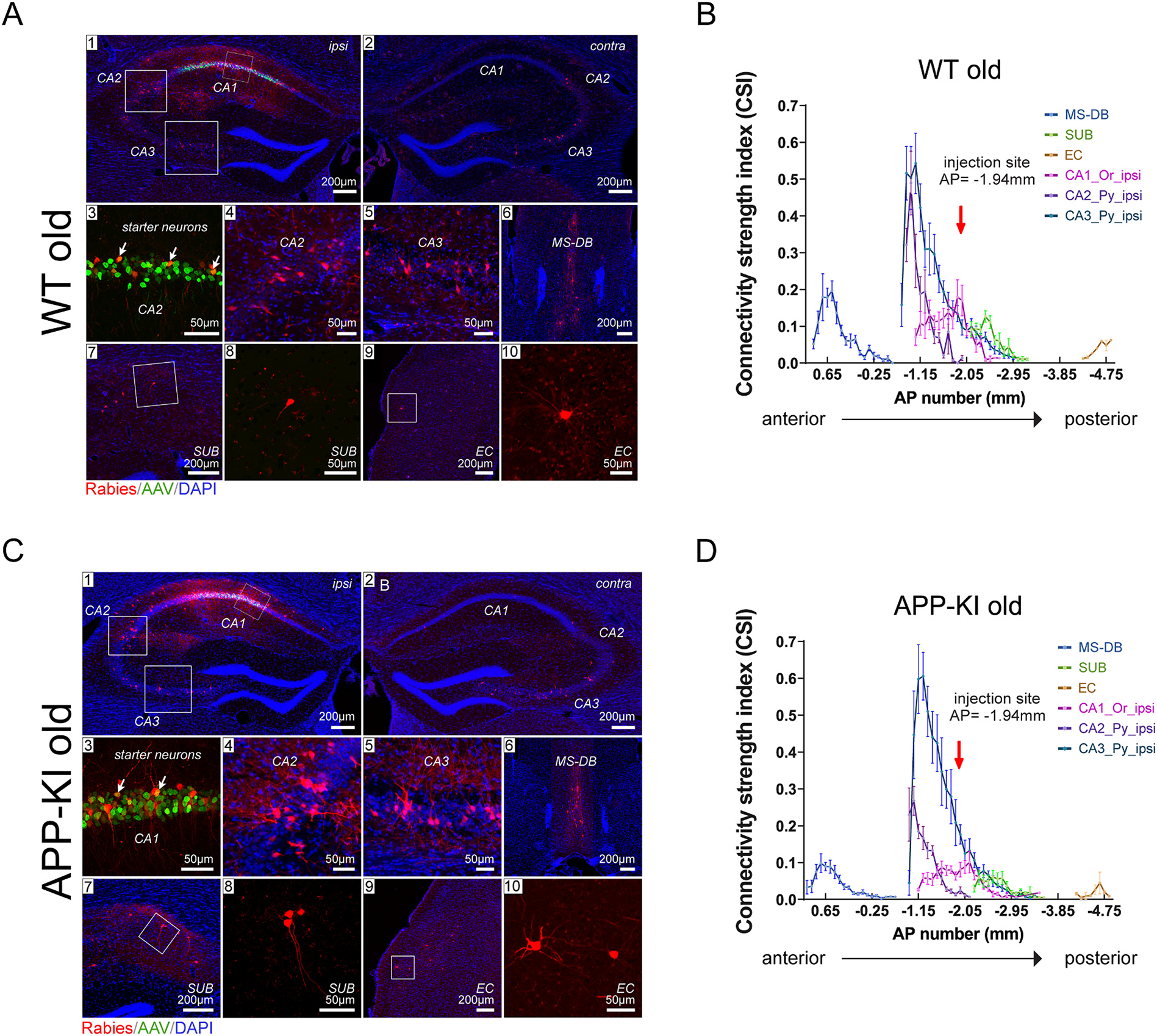
Monosynaptic rabies tracing maps specific regional inputs to excitatory CA1 cells along the anterior-posterior (AP) axis in old WT and APP-KI mice. A, C) Representative fluorescent coronal section images from WT old (n = 10) and APP-KI old (n = 8), respectively. Rabies virus-infected neurons are labeled by DsRed, and AAV-infected neurons are in green. All slices are counterstained by DAPI in blue. For both A and C, (1) shows the ipsilateral hippocampal formation including the CA1 injection site. (2), rabies virus mapped presynaptic inputs in the contralateral hippocampus. (3), enlarged image of CA1 starter neurons expressing both EGFP and DsRed fluorescent proteins from AAV and rabies virus. For both A and C (4–10), results of rabies virus-mediated retrograde monosynaptic tracing from CA1. The input regions include hippocampal CA2 (4), CA3 (5), medial septum and diagonal band (MS-DB) (6), subiculum (SUB) (7 and 8), and entorhinal cortex (EC) (9 and 10). (3), (4), (5), (8), and (10) are enlarged views of the white boxed areas shown in (1), (7), and (9), respectively. Scale bars are labeled for each panel. B, D) The connectivity strength index (CSI) distribution along AP positions across the whole brain. CSI is defined as the number of input neurons normalized by the number of starter neurons; AP position is given relative to bregma values. The red arrow at AP = −1.94 mm shows the position of the injection site. Representative input regions were used for the AP plot, including the ipsilateral hippocampal CA1 oriens layer (Or), ipsilateral CA2 and ipsilateral CA3 pyramidal layers (Py), as well as MS-DB, SUB, and EC. (For interpretation of the references to colour in this figure legend, the reader is referred to the web version of this article.)

We focused on mapping hippocampal CA1 excitatory neurons because these cells operate as place cells and are directly relevant to spatial mapping behaviors that are impaired during AD progression (Soltesz and Losonczy, 2018). To map the global and local neural circuit connections of CA1 excitatory neurons in AD mice, we performed Cre-dependent monosynaptic rabies tracing in age-matched young and old APP-KI and WT mice as shown schematically (Fig. 2A). The rabies tracing experiment was performed at 3–4 months and 10–11 months old as specified above (Fig. 1). A mixture of helper AAVs (AAV-DIO-TC66T-GFP-oG and AAV-CaMKIIα-EGFP-Cre virus) was injected at the pyramidal layer of hippocampal CA1 in the left hemisphere of the mouse brains for each age and genotype noted above. The Cre-dependent AAV helper (AAV-DIO-TC66T-GFP-oG) expresses TC66T, a variant of tumor virus receptor A (TVA) which acts as a high specific target that facilitates rabies infection, and optimized glycoprotein (oG) (Miyamichi et al., 2013). The Cre recombinase under the CaMKIIα promoter AAV (AAV-CaMKIIα-EGFP-Cre) directs the expression of Cre recombinase to CaMKIIα positive excitatory neurons. The combination of DIO (double-floxed inverted open reading frame) and Cre provided by the two AAVs restrict TC66T and oG expression in CaMKIIα positive excitatory neurons. At 17 days after the primary AAV helper viral injection, the EnvA pseudotyped G deleted rabies virus (EnvA-SADΔG-DsRed) was delivered at the same injection site as the AAV injection (Fig. 2A) as guided by stereotaxis coordinates. Mouse brains were harvested 9 days after the second injection for sectioning and further histological processing. Following the monosynaptic rabies virus tracing from CA1 starter neurons, the input-mapped presynaptic neurons are primarily located in the MS-DB, hippocampal CA1, CA2, CA3, subiculum (SUB), and entorhinal cortex (EC), as shown in the schematic (Fig. 2B). To compare potential connectivity differences between young and old WT and APP-KI mice, brain-wide coronal sections in WT young, WT old, APP-KI young and APP-KI old mice were imaged and quantified. The overall numbers of presynaptic neurons were quantified and normalized by the total starter neurons of each case; the resultant brain-wide overall connectivity was compared across the groups of mice. The expression of the rabies virus (EnvA-SADΔG-RV-DsRed) is visualized with DsRed, and the expression of the helper AAV is visualized with EGFP. While presynaptic neurons are only labeled with DsRed from the RV gene expression, the starter cells can be unambiguously identified by their EGFP and DsRed expression from both the helper AAV and ΔG-DsRed rabies virus (Fig. 3A; Fig. 4A). APP-KI young mice (*n* = 10) show significantly weaker brain-wide overall connectivity relative to WT young mice (*n* = 7) (WT young: 22.10 ± 1.97, APP-KI young: 14.41 ± 0.72, Wilcoxon rank-sum test, *p* = 2.0 × 10^−3^). Similarly, APP-KI old mice (*n* = 8) also show weaker overall connectivity relative to WT old mice (n = 10) (Fig. 2C) (WT old: 21.01 ± 2.14, APP-KI old: 14.36 ± 1.92, Wilcoxon rank-sum test, *p* = 3.40 × 10^−2^). Our data indicate that the CA1 excitatory neurons input connections in APP-KI mice show compromised brain-wide connectivity and they are overall weaker relative to those of age-matched WT mice.

### 3.3. Regional-specific tracing results in age-matched young and old WT and APP-KI mice

To map and compare regional-specific presynaptic inputs to CA1 excitatory cells in WT young, APP-KI young, WT old and APP-KI old mice, representative coronal sections of ipsilateral and contralateral hippocampal formations, injection sites and presynaptic inputs regions are imaged and quantified (WT young: Fig. 3A and B; APP-KI young: Fig. 3C and D; WT old: Fig. 4A and B; APP-KI old: Fig. 4C and D). Neurons labeled with both red (DsRed) and green (GFP) fluorescence signals represent the robust labeling of starter neurons (Fig. 3A and C, Fig. 4A and C, panel 3) and their numbers are used as the denominators for semi-quantitative connectivity strength measurements. Neurons labeled with DsRed only are the presynaptic inputs of starter neurons. For both WT and APP-KI mice, the prominent input regions to excitatory CA1 pyramidal neurons include the medial septum-diagonal band (MS-DB) complex, CA3 and CA2 pyramidal (Py) layer, SUB, and EC (Fig. 3A and C, Fig. 4A and C, panel 1–10). Other CA1 excitatory neuron inputs include putative inhibitory interneurons located in the oriens (Or), radiatum (Rad) and lacunosum moleculare (Lmol) layer of hippocampal CA1, CA2 and CA3, as well as median raphe, paramedian raphe (MnR/PMnR), and sparse labeling in the nucleus reuniens. The greatest input density comes from the ipsilateral side of the brain, but robust contralateral inputs can be detected from hippocampal subregions: CA1-3 (Fig. 3A and C, Fig. 4A and C).

To investigate the distribution of CA1 circuit inputs along the anterior-to-posterior (AP) axis in the brains for WT young, APP-KI young, WT old and APP-KI old groups, the input connectivity strengths index measurements of selected presynaptic input regions are plotted across AP locations of individual brain sections. The brain regions include MS-DB, SUB, EC, ipsilateral CA1 oriens layer, ipsilateral CA2 pyramidal layer and ipsilateral CA3 pyramidal layer (Fig. 3B and D, Fig. 4B and D). CA1 excitatory cells in the four groups of mice show an overall similar distribution pattern of regional inputs, with MS-DB accounting for the most anterior position, followed by the hippocampal formation, including CA1, CA2, CA3, SUB, and finally EC, moving progressively more posteriorly.

To quantitatively compare the connectivity strength of CA1 excitatory neurons in age-matched WT and APP-KI mice, the presynaptic input neurons and starter neurons were identified and counted throughout each brain. To determine semi-quantitative normalized neural connectivity, the connectivity strength index (CSI) was calculated for each pair of connected brain subregions, represented by the number of neurons within a specific subregion over the total number of starter neurons in each case.

As seen in the plots of all four groups of mice tested, there is a sharp peak of CA3 input strength in the CA3 locations anterior to the CA1 injection site, as the anatomical structure of CA3 starts more anterior as compared to CA1 (Fig. 3B and D, Fig. 4B and D). Among these regions, the ipsilateral CA3 Py layer provides the greatest number of inputs, as shown by the largest area-under-curve. However, there is a significant decrease in area-under-curve for both MS-DB (WT young vs APP-KI young, *p* = 0.0002; WT old vs APP-KI old, *p* = 0.0001; paired Wilcoxon rank sum test) and ipsilateral CA1 Oriens (WT young vs APP-KI young, *p* < 0.00001; WT old vs APP-KI old, *p* = 0.0012; paired Wilcoxon rank sum test) inputs in APP-KI mice compared to WT mice for both age groups.

**Fig. 5.**
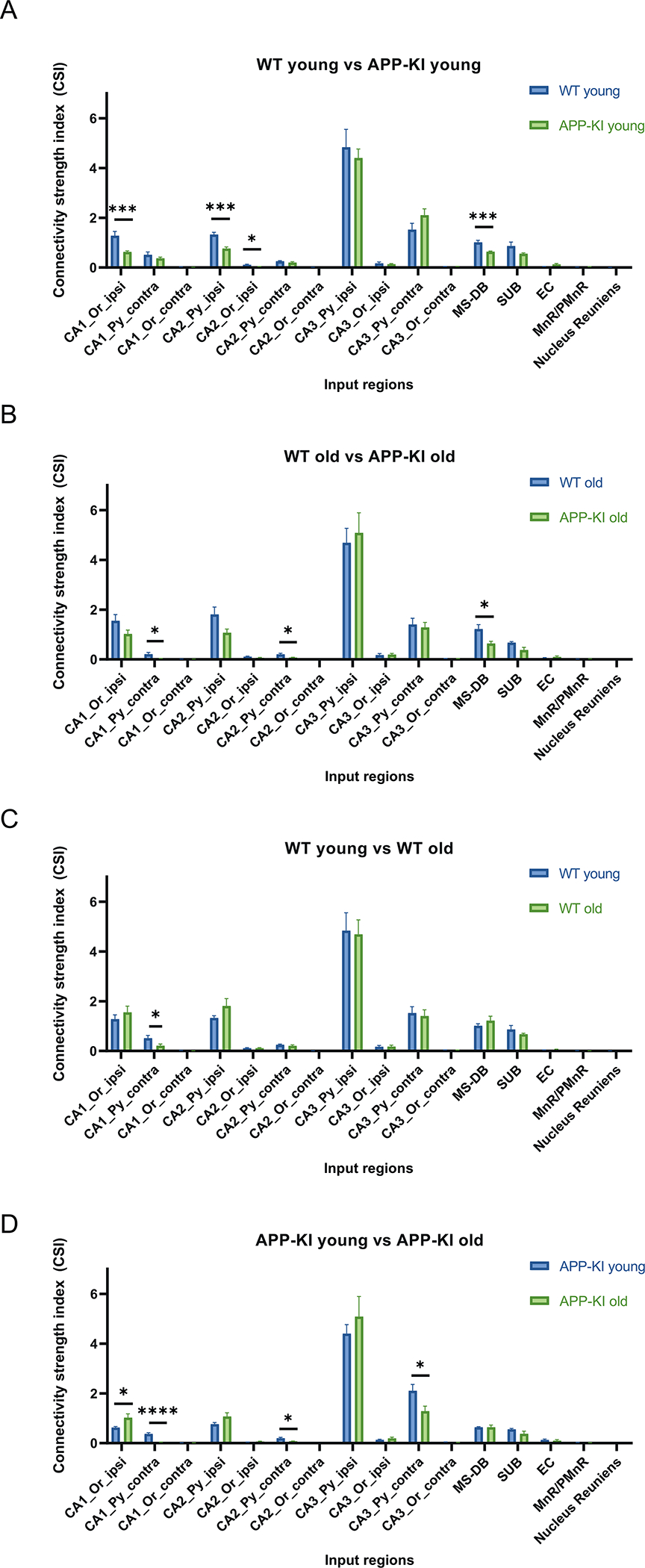
Connectivity strength index (CSI) quantification of presynaptic input regions. Quantitative analysis of CA1 CSI values across WT young and old mice and APP-KI young and old mice. The CSI is the ratio of the CA1 input neuron number in a subregion to the total starter neuron number in a brain. The input regions include hippocampal CA1, CA2, and CA3 oriens layer (or) and pyramidal layer (Py), from both the ipsilateral and contralateral sides of the CA1 injection site, as well as the MS-DB, SUB, and EC. A) WT young mice (n = 7) have significantly higher CSI values relative to APP-KI young mice (n = 10) in the CA1_Or_ipsi, CA2_Py_ipsi, CA2_Or_ipsi, and MS-DB regions (Wilcoxon rank-sum test, *p* = 4.11 × 10^−4^, p = 4.11 × 10^−4^, *p* = 1.36 × 10^−2^, *p* = 1.03 × 10^−4^, respectively). B) WT old mice (n = 10) have significantly higher CSI values relative to APP-KI old mice (n = 8) in the CA1_Py_contra, CA2_Py_contra, and MS-DB regions (Wilcoxon rank-sum test, *p* = 2.64 × 10^−2^, *p* = 1.17 × 10^−2^, *p* = 1.55 × 10^−2^, respectively). C) WT young mice have significantly higher CSI values relative to WT old mice in the CA1_Py_contra region (Wilcoxon rank-sum test, *p* = 2.34 × 10^−2^). D) APP-KI old mice have significantly higher CSI values relative to APP-KI young mice in the CA1_Or_ipsi region (Wilcoxon rank-sum test, p = 1.17 × 10^−2^). APP-KI young mice have significantly higher CSI values relative to APP-KI old mice in the CA1_Py_contra, CA2_Py_contra, and CA3_Py_contra regions (Wilcoxon rank-sum test, *p* = 4.6 × 10^−5^, p = 1.55 × 10^−2^, *p* = 4.34 × 10^−2^, respectively). * p < 0.05, ** p < 0.01, *** p < 0.001, **** *p* < 0.0001. All data are represented with mean ± SEM. See also Supplementary Tables S1 and S2.

As expected, the majority of CA1 input connections originate within the hippocampal formation. Overall, ipsilateral hippocampal inputs are much more abundant relative to contralateral inputs (Fig. 5; Supplemental Tables 1 and 2). For all contralateral inputs, the contralateral CA3 pyramidal layer has the highest density of inputs, indicating a strong commissural CA3 to CA1 pathway. Detailed statistical comparisons between the four groups of mice reveal significant CSI differences (Fig. 4, Supplemental Tables 1 and 2). Comparing age-matched WT and APP-KI mice, ipsilateral CA1 Or, ipsilateral CA2 Py, ipsilateral CA2 Or and MS-DB of the APP-KI young group all show significantly weaker input connections to excitatory CA1 neurons relative to the WT young group (Fig. 5A) (Wilcoxon rank-sum test, *p* = 4.11 × 10^−4^, p = 4.11 × 10^−4^, *p* = 1.36 × 10^−2^, *p* = 1.03 × 10^−4^, respectively). The CSI values of WT young group are 1.281 ± 0.172 (CA1_Or_ipsi), 1.329 ± 0.086 (CA2_Py_ipsi), 0.107 ± 0.029 (CA2_Or_ipsi), 1.011 ± 0.087 (MS-DB); the CSI values of APP-KI young group are 0.626 ± 0.041 (CA1_Or_ipsi), 0.763 ± 0.062 (CA2_Py_ipsi), 0.037 ± 0.007 (CA2_Or_ipsi), 0.640 ± 0.024 (MS-DB). For comparison between the two old age groups, APP-KI old mice show significantly smaller CSI values in contralateral CA1 Py, contralateral CA2 Py and MS-DB compared to WT old mice (Fig. 5B) (Wilcoxon rank-sum test, *p* = 2.64 × 10^−2^, *p* = 1.17 × 10^−2^, *p* = 1.55 × 10^−2^, respectively). The CSI values of WT old group are 0.211 ± 0.073 (CA1_Py_contra), 0.206 ± 0.040 (CA2_Py_contra), 1.220 ± 0.180 (MS-DB); the CSI values of APP-KI old group are 0.031 ± 0.012 (CA1_Py_contra), 0.076 ± 0.013 (CA2_Py_contra), 0.638 ± 0.090 (MS-DB). In terms of comparison within the same genotype but at different ages, WT young and WT old groups show a significant difference at contralateral CA1 Py (Fig. 5C) (Wilcoxon rank-sum test, *p* = 2.34 × 10^−2^). Their CSI values are 0.511 ± 0.112 (WT young group) and 0.211 ± 0.073 (WT old group), showing decreased connectivity in old WT mice. Age-related connectivity differences are more frequently observed in APP-KI mice. For APP-KI young and APP-KI old mice, contralateral CA1 Py, contralateral CA2 Py and contralateral CA3 Py all show significantly weaker connections in APP-KI old relative to APP-KI young (Fig. 5D) (Wilcoxon rank-sum test, *p* = 4.6 × 10^−5^, p = 1.55 × 10^−2^, *p* = 4.34 × 10^−2^, respectively). The CSI values of APP-KI young group are 0.370 ± 0.046 (CA1_Py_contra), 0.198 ± 0.040 (CA2_Py_contra), 2.103 ± 0.257 (CA3_Py_contra); the CSI values of APP-KI old group are 0.031 ± 0.012 (CA1_Py_contra), 0.076 ± 0.013 (CA2_Py_contra), 1.287 ± 0.200 (CA3_Py_contra). Unexpectedly, there is an increase in the CSI value of the ipsilateral CA1 oriens layer when comparing APP-KI young vs APP-KI old (Wilcoxon rank-sum test, *p* = 1.17 × 10^−2^). Their CSI values are 0.626 ± 0.041 (APP-KI young group) and 0.947 ± 0.152 (APP-KI old group). Together, these data support significant alterations in presynaptic connectivity strength to CA1 excitatory neurons in APP-KI mice compared to WT mice. Moreover, these connectivity alterations show age-progressive features in WT and especially in APP-KI mice.

### 3.4. Quantitative analysis of the regional-specific proportions of CA1 excitatory cell input pattern

**Fig. 6.**
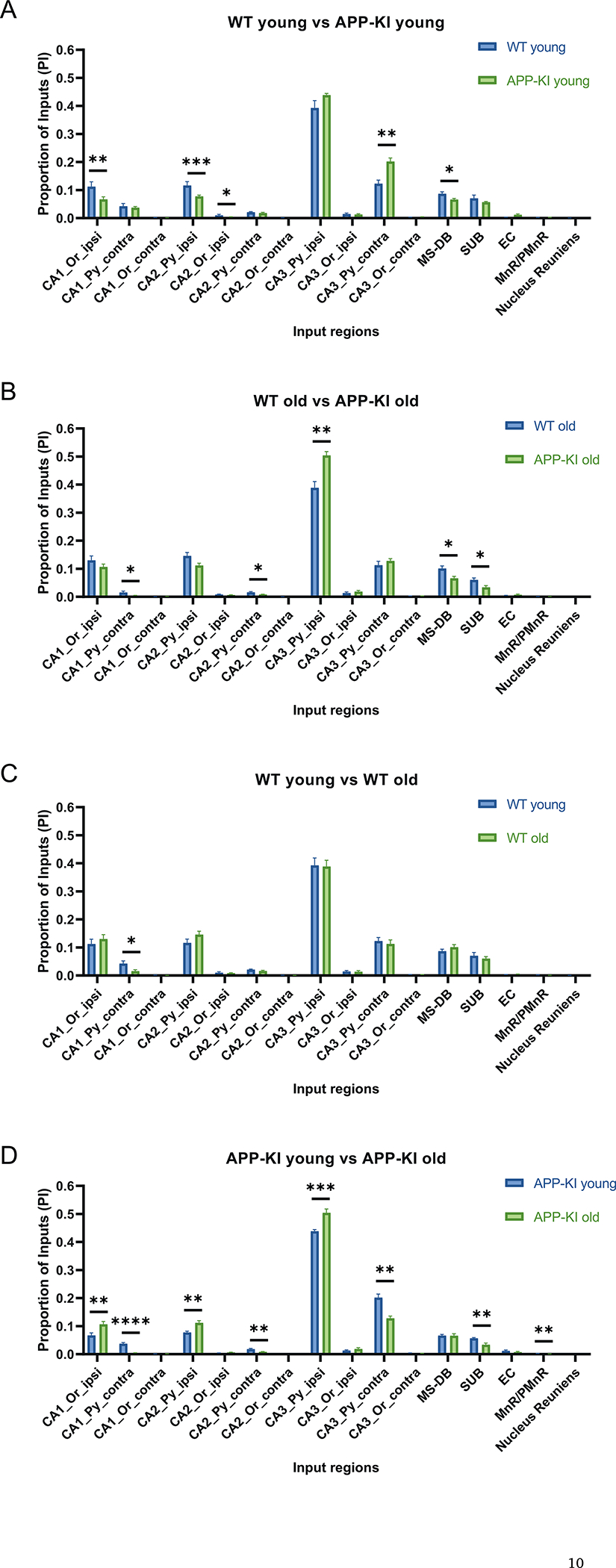
Proportion of inputs (PI) quantification of presynaptic input regions. Quantitative analysis of PI values across four groups of mice. The PI is the ratio of the input neuron number in a subregion to the total input neuron number in a brain. The input regions include hippocampal CA1, CA2, and CA3 oriens layer (Or) and pyramidal layer (Py), from both the ipsilateral and contralateral sides of the injection site, as well as the MS-DB, SUB, and EC. A) APP-KI young mice (n = 10) have significantly lower PI values relative to WT young mice (n = 7) in the CA1_Or_ipsi, CA2_Py_ipsi, CA2_Or_ipsi and MS-DB regions (Wilcoxon rank-sum test, *p* = 4.63 × 10^−3^, p = 4.11 × 10^−4^, *p* = 1.85 × 10^−2^, *p* = 2.50 × 10^−2^, respectively). APP-KI young mice have significantly higher PI values relative to WT young mice in the CA3_Py_contra region (Wilcoxon rank-sum test, *p* = 1.95 × 10^−3^). B) APP-KI old mice (n = 8) have significantly lower PI values relative to WT old mice (n = 8) in the CA1_Py_contra, CA2_Py_contra, MS-DB and SUB regions (Wilcoxon rank-sum test, *p* = 3.39 × 10^−2^, *p* = 2.66 × 10^−2^, p = 1.17 × 10^−2^, p = 1.55 × 10^−2^, respectively). APP-KI old mice have significantly higher PI values relative to WT old mice in the CA3_Py_ipsi region (Wilcoxon rank-sum test, *p* = 1.37 × 10^−3^). C) WT young mice have significantly higher PI values relative to WT old mice in the CA1_Py_contra region (Wilcoxon rank-sum test, p = 1.36 × 10^−2^). D) APP-KI old mice have significantly higher PI values relative to APP-KI young mice in the CA1_Or_ipsi, CA2_Py_ipsi regions, and CA3_Py_ipsi (Wilcoxon rank-sum test, *p* = 2.06 × 10^−3^, *p* = 3.06 × 10^−3^, *p* = 3.2 × 10^−4^, respectively). APP-KI young mice have significantly higher PI values relative to APP-KI old mice in the CA1_Py_contra, CA2_Py_contra, CA3_Py_contra, SUB and MnR/PMnR regions (Wilcoxon rank-sum test, p = 4.6 × 10^−5^, *p* = 6.22 × 10^−3^, p = 3.06 × 10^−3^, p = 3.06 × 10^−3^, *p* = 8.48 × 10^−3^, respectively). All data are represented with mean ± SEM. * p < 0.05, ** p < 0.01, *** p < 0.001, **** p < 0.0001. See also Supplementary Tables S1 and S2.

To investigate whether the CA1 input connectivity patterns shift in APP-KI mice with their overall reduced connectivity, we measured the proportion of input (PI) index based on the proportion of each input region for the total inputs (Fig. 6). The PI index is represented by the fraction of presynaptic neurons in each input region over the entire brain inputs and provides a more global measure of connectivity contribution for a given region. For comparison between WT young and APP-KI young mice, the APP-KI young group shows significantly lower PI values in the ipsilateral CA1 oriens layer, ipsilateral CA2 pyramidal layer, ipsilateral CA2 oriens layer, and MS-DB (Fig. 6A) (Wilcoxon rank-sum test, *p* = 4.63 × 10^−3^, *p* = 4.11 × 10^−4^, *p* = 1.85 × 10^−2^, *p* = 2.50 × 10^−2^, respectively). The PI values of WT young group are 0.113 ± 0.017 (CA1_Or_ipsi), 0.117 ± 0.014 (CA2_Py_ipsi), 0.010 ± 0.004 (CA2_Or_ipsi), 0.087 ± 0.007 (MS-DB); the PI values of APP-KI young group are 0.067 ± 0.009 (CA1_Or_ipsi), 0.078 ± 0.005 (CA2_Py_ipsi), 0.004 ± 0.001 (CA2_Or_ipsi), 0.067 ± 0.004 (MS-DB). In contrast, the contralateral CA3 pyramidal layer shows significantly higher PI for young APP-KI mice relative to young WT mice (Fig. 6A) (Wilcoxon rank-sum test, *p* = 1.95 × 10^−3^). The PI value of the WT young group is 0.123 ± 0.013 (CA3_Py_contra) and the PI value of the APP-KI young group is 0.202 ± 0.013 (CA3_Py_contra). The unexpected increase in hippocampal connectivity might reflect compensatory increases in parts of the hippocampal formation in response to circuit input decreases in other parts of the hippocampus. For comparisons between WT old and APP-KI old groups, APP-KI old group shows lower PI values in the contralateral CA1 pyramidal layer, contralateral CA2 pyramidal layer, MS-DB and SUB (Fig. 6B) (Wilcoxon rank-sum test, *p* = 3.39 × 10^−2^, *p* = 2.66 × 10^−2^, *p* = 1.17 × 10^−2^, *p* = 1.55 × 10^−2^, respectively). The PI values of the WT old group are 0.016 ± 0.005 (CA1_Py_contra), 0.016 ± 0.003 (CA2_Py_contra), 0.101 ± 0.009 (MS-DB), 0.061 ± 0.007 (SUB); the PI values of the APP-KI old group 0.004 ± 0.002 (CA1_Py_contra), 0.008 ± 0.001 (CA2_Py_contra), 0.066 ± 0.007 (MS-DB), 0.034 ± 0.006 (SUB). In contrast, the ipsilateral CA3 pyramidal layer is higher in APP-KI old group than WT old group (Fig. 6B) (Wilcoxon rank-sum test, *p* = 1.37 × 10^−3^). Their PI values are 0.389 ± 0.023 (CA3_Py_ipsi, WT old group) and 0.504 ± 0.014 (CA3_Py_ipsi, APP-KI old group). A significant difference was observed in the comparison between WT young and WT old, where the PI value of contralateral CA1 pyramidal layer is smaller in WT old relative to in WT young group (Fig. 6C) (WT young: 0.042 ± 0.010, WT old: 0.016 ± 0.005, Wilcoxon rank-sum test, *p* = 1.36 × 10^−2^). Unexpectedly, for age-related comparison between APP-KI young and APP-KI old, the inputs from ipsilateral CA1 oriens layer, ipsilateral CA2 pyramidal layer, and ipsilateral CA3 pyramidal layer all show higher PI values in the APP-KI old group (Fig. 6D) (Wilcoxon rank-sum test, *p* = 2.06 × 10^−3^, *p* = 3.06 × 10^−3^, *p* = 3.2 × 10^−4^, respectively). The PI values of APP-KI young group are 0.067 ± 0.009 (CA1_Or_ipsi), 0.078 ± 0.005 (CA2_Py_ipsi), 0.438 ± 0.006 (CA3_Py_ipsi); the PI values of APP-KI old group are 0.107 ± 0.011 (CA1_Or_ipsi), 0.112 ± 0.008 (CA2_Py_ipsi), 0.504 ± 0.014 (CA3_Py_ipsi). By comparison, the contralateral CA1 pyramidal layer, contralateral CA2 pyramidal layer, contralateral CA3 pyramidal layer, SUB, and MnR/PMnR all show lower proportions of inputs in the APP-KI old group (Fig. 6B) (Wilcoxon rank-sum test, *p* = 4.6 × 10^−5^, *p* = 6.22 × 10^−3^, p = 3.06 × 10^−3^, p = 3.06 × 10^−3^, *p* = 8.48 × 10^−3^, respectively). The PI values of APP-KI young group are 0.037 ± 0.004 (CA1_Py_contra), 0.018 ± 0.003 (CA2_Py_contra), 0.202 ± 0.013 (CA3_Py_contra), 0.057 ± 0.003 (SUB), 0.0027 ± 0.0003 (MnR/PMnR); the PI values of APP-KI old group are 0.004 ± 0.001 (CA1_Py_contra), 0.008 ± 0.001 (CA2_Py_contra), 0.128 ± 0.008 (CA3_Py_contra), 0.034 ± 0.006 (SUB), 0.0011 ± 0.0004 (MnR/PMnR). Together, these data support the idea that compensatory circuit input changes may occur in response to connectivity losses in AD conditions.

### 3.5. Sex differences in connectivity strength and pattern to CA1 excitatory neurons

In human patients, AD is more common in females than males on an age-matched basis (Seshadri et al., 1997). We asked whether sex differences in CA1 neuron connectivity strength and patterns may be recapitulated in the AD mouse model. The CSI and PI values were compared between male and female mice for each group pair. Notably, for both CSI and PI, aged female APP-KI mice show significant connectivity defects for the SUB to CA1 projection as compared to aged male APP-KI mice (Wilcoxon rank-sum test, CSI *p* = 3.6 × 10^−2^, PI p = 3.6 × 10^−2^), while no significant differences are detected for other input regions when comparing between sexes. These results indicate that the circuit connections to CA1 excitatory neurons from different brain regions may be differentially impacted by sex-related factors.

## 4. Discussion

In this study, we applied monosynaptic rabies tracing approach to investigate alterations in input circuit connectivity of hippocampal CA1 excitatory neurons in age-matched WT and APP-KI mice. As immunostaining results show age-progressive amyloid deposition in hippocampal CA1 in APP-KI mice, our monosynaptic rabies virus tracing reveals pathological impairments of excitatory CA1 cell input connectivity. We map the local and long-range presynaptic inputs to CA1 excitatory neurons from multiple brain regions in age-matched WT and APP-KI mouse groups at young and old ages. Compared to WT mice, AD mice show an overall impaired hippocampal CA1 circuit connectivity. Our results show age-progressive and reduced hippocampal CA1 connectivity strength in APP-KI mice compared to WT mice, as well as interesting connectivity patterns shifts with different hippocampal regions.

Quantification of the WT and APP-KI mice in young and old age groups reveals age-associated AD circuit change in both long-range and local brain regions. For example, presynaptic inputs from MS-DB in APP-KI mice compared to WT mice are weaker in both age groups. MS-DB is an essential modulator of hippocampal activity (Roland et al., 2014). Accumulating evidence shows that the septal regions are involved in AD. Cholinergic neurons in MS are found to decrease substantially in AD patients and cholinergic innervations to the hippocampus are severely impacted (Hampel et al., 2018; Nelson et al., 2014; Takeuchi et al., 2021). Previous CA1 excitatory studies show that most of the rabies-labeled septohippocampal cells were cholinergic (Sun et al., 2014). The decrease of MS-DB to CA1 excitatory neuron connectivity revealed by monosynaptic rabies tracing may reflect a cholinergic neural connectivity strength decrease in AD mice.

It is noteworthy that local intrahippocampal connectivity changes with genotype and age. Surprisingly, the connectivity strength input proportional pattern of CA3 pyramidal neurons is significantly larger in APP-KI old mice relative to WT old mice and is also higher in APP-KI old mice compared to APP-KI young mice. Similarly, there is a trend of increase in CA3 to CA1 connectivity in APP-KI young when compared to WT young group (Fig. 6A). The major connections to CA1 excitatory neurons originate from the CA3 pyramidal neurons. While the absolute CSI value of CA3 pyramidal neurons to CA1 excitatory neurons shows no significant difference when comparing different ages and genotypes, the proportional input pattern shifts significantly in APP-KI compared to WT mice. This raises the interesting possibility that there may be a compensatory effect of CA1 neural plasticity during APP progression. A previous study has shown that DG to CA3 GABAergic transmission and feedforward inhibition is reduced in CA3 pyramidal neurons of APP/PS1 mice (Viana da Silva et al., 2019). CA1 neurons receive robust information from CA3 neurons through the trisynaptic pathway. It is possible that the decrease of DG-CA3 inhibition leads to the hyperexcitation of CA3 neurons, and further contributes to the enhancement of the CA3 to CA1 pathway. Moreover, *in vivo* electrophysiological study discovered that APP-KI mice exhibit disrupted spatial remapping of CA1 place cells (Jun et al., 2020). Their results suggest that MEC → CA1 signal transfer via fast gamma oscillations is deteriorated in APP-KI mice, whereas slow gamma-mediated CA3 → CA1 signal transfer remains relatively intact. The compromised remapping of CA1 neurons may indicate a potential differential impact on EC-CA1 and CA3-CA1 neural communication. We also note that the connectivity strength of contralateral CA1 pyramidal neuron inputs to ipsilateral CA1 excitatory neuron decreases in aged groups. This may reflect an age-dependent progressive deterioration of commissural inputs.

The age-progressive Aβ deposition in CA1 and the overall compromised connectivity of CA1 neurons in APP-KI mice, together, indicate that Aβ level may be associated with attenuated synaptic connections, as synaptic loss is a hallmark of AD. Increasing evidence has shown that Aβ deposition affects both local and long-range circuit connections in the brain. At the very early stage of the disease, Aβ induces neuron hyperactivity and excitatory/inhibitory imbalance by decreasing inhibitory GABAergic function (Busche et al., 2008) and causes glutamate accumulation near the synapse (Zott et al., 2019). As the disease progresses, the prolonged Aβ depositions further reduce glutamatergic synaptic transmission and lead to synaptic loss (Calvo-Rodriguez and Bacskai, 2021; Palop and Mucke, 2010). Three-dimensional analysis of hippocampal CA1 brain samples from AD human postmortem tissue shows that the total number of synapses is reduced at the early stages of AD, and this reduction progressively increases in severity during the late stage of the disease (Montero-Crespo et al., 2021). This age-related synaptic deterioration has functional implications. *In vivo* two-photon Ca^2+^ imaging of hippocampal CA1 in an AD model mouse reveals that in the vicinity of plaque, CA1 pyramidal neuron activity is profoundly impacted, especially in aged AD mice (Busche et al., 2012). These earlier relevant functional and physiological studies support the potential functional consequences of AD-impacted neural circuit connectivity in hippocampal CA1 in our present study.

Our results also indicate sex differences in neural circuit connectivity to hippocampal CA1 in AD model mice, as more severe SUB-CA1 projection defects are found in female old APP-KI mice than in the male. This result is consistent with the well-established finding that there is a two-fold increased risk of AD in women versus men (Seshadri et al., 1997). As the SUB-CA1 connections have been implicated in object-location memory (Sun et al., 2019) and loss of object location memory is one of the key impairments in AD, our new finding in hippocampal sub-circuit mechanisms may provide an intriguing new target to counteract AD-related memory impairments.

The method of rabies labeling is sensitive and reliable, because labeled cells are seen in very distant structures such as the MS-DB area and other areas that are known to project weakly to hippocampal CA1. However, we do not expect this method to label every input to each neuron with 100% efficiency. The connectivity from EC to CA1 measured in this study is relatively sparse compared to earlier studies with bulk tracer injections with no quantitative measurements of project strengths. Note that our rabies viral tracing approach offers an important technical advantage so that we can quantitatively assess the relative number of inputs from each source to each target cell type, and quantify the number of cells that are labeled at various input locations, thus providing weighted connection strengths for defined cell types. The EC-CA1 connectivity strength index (CSI) value measured in this study is CSI = 0.034 ± 0.011 for WT young group (*n* = 7, 3–4 months), and CSI = 0.052 ± 0.022 for WT old group (*n* = 10, 8–11 months). These values are comparable to the CSI value reported in a previous CA1 excitatory neuron rabies tracing study (Sun et al., 2014). Since we are quantitatively investigating the circuit connectivity differences between WT and APP-KI mice at young and old ages using the same experimental strategy, the results for comparison across different groups are reliable, and are supported in general based on our earlier published results.

Together, our research showing the connectivity strength alterations and connectivity pattern shifts in APP-KI AD mice may provide insights for tackling AD at the neural circuit level. Future neural circuit studies may lead to improved therapeutic interventions that slow down or counteract this disease at an early stage in AD patients.

## 5. Conclusions

In conclusion, we applied Cre-dependent monosynaptic rabies tracing to study the circuit connectivity changes of hippocampal CA1 excitatory neurons in age-matched WT and APP-KI mice. Using semi-quantitative analysis, we identify age-progressive connectivity strength changes of CA1 neurons in AD model mice. Interestingly, our data show compensatory changes in CA3-CA1 connectivity patterns in AD model mice, as well as sex differences in SUB-CA1 connections.

## Supplementary Material

Supplement

## Abbreviations:

AD: Alzheimer’s disease
APP-KI: amyloid precursor protein gene knock-in mouse model
Aβ: amyloid-beta
AAV: adeno-associated virus
AP: anteroposterior
ML: mediolateral
DV: dorsoventral
WT: wild type
PBS: phosphate-buffered saline
PFA: paraformaldehyde
CaMKII: calcium/calmodulin-dependent kinase II
FOV: field of view
CSI: connectivity strength index
PI: proportion of input
Ipsi: ipsilateral
Contra: contralateral
Py: pyramidal
Or: oriens
Rad: radiatum
Lmol: lacunosum moleculare
MS-DB: medial septum and diagonal band
SUB: subiculum
EC: entorhinal cortex
MnR/PMnR: median raphe/paramedian raphe
