# Supplementary material for: Hippocampal neural circuit connectivity alterations in an Alzheimer’s disease mouse model revealed by monosynaptic rabies virus tracing
Source: Neurobiol Dis. Author manuscript; Available in PMC 2022 Oct 1. (PMC9482455; doi:10.1016/j.nbd.2022.105820)
Supplement: Supplement [file NIHMS1834490-supplement-Supplement.pdf]

Figure

Click here to access/download;Figure;Figures_NBD_round1_07062022-

Supplementary Table 1.
Data Summary of Connectivity Strength Index (CSI) and Proportion of Inputs (PI) Measurements

CSI Summary	WT young		WT old		APP-KI young		APP-KI old	
	Mean	SEM	Mean	SEM	Mean	SEM	Mean	SEM
CA1_or_ipsi	1.2809	0.1721	1.5535	0.2512	0.6259	0.0410	0.9465	0.1517
CA1_py_contra	0.5111	0.1124	0.2108	0.0734	0.3701	0.0456	0.0309	0.0123
CA1_or_contra	0.0187	0.0053	0.0091	0.0033	0.0115	0.0024	0.0117	0.0047
CA2_py_ipsi	1.3293	0.0858	1.8069	0.2971	0.7633	0.0621	1.0734	0.1448
CA2_or_ipsi	0.1070	0.0291	0.1072	0.0276	0.0372	0.0072	0.0622	0.0165
CA2_py_contra	0.2496	0.0257	0.2061	0.0400	0.1975	0.0398	0.0764	0.0126
CA2_or_contra	0.0060	0.0060	0.0022	0.0016	0.0031	0.0012	0.0014	0.0014
CA3_py_ipsi	4.8377	0.7154	4.6866	0.5800	4.4030	0.3643	5.0838	0.8064
CA3_or_ipsi	0.1711	0.0534	0.1769	0.0569	0.1329	0.0232	0.1905	0.0473
CA3_py_contra	1.5206	0.2597	1.4033	0.2529	2.1028	0.2571	1.2865	0.2000
CA3_or_contra	0.0333	0.0116	0.0295	0.0109	0.0402	0.0129	0.0258	0.0072
MS-DB	1.0107	0.0869	1.2202	0.1799	0.6404	0.0236	0.6379	0.0897
SUB	0.8626	0.1605	0.6709	0.0446	0.5553	0.0360	0.3799	0.1044
EC	0.0339	0.0109	0.0516	0.0218	0.1277	0.0354	0.0984	0.0394
MnR/PMnR	0.0188	0.0047	0.0084	0.0048	0.0267	0.0036	0.0151	0.0063
Nucleus Reuniens	0.0044	0.0029	0.0000	0.0000	0.0022	0.0007	0.0011	0.0011

PI Summary	WT young		WT old		APP-KI young		APP-KI old	
	Mean	SEM	Mean	SEM	Mean	SEM	Mean	SEM
CA1_or_ipsi	0.1123	0.0170	0.1302	0.0157	0.0670	0.0094	0.1066	0.0105
CA1_py_contra	0.0424	0.0096	0.0158	0.0049	0.0373	0.0043	0.0039	0.0015
CA1_or_contra	0.0015	0.0005	0.0007	0.0002	0.0010	0.0002	0.0013	0.0005
CA2_py_ipsi	0.1165	0.0135	0.1458	0.0121	0.0776	0.0046	0.1116	0.0084
CA2_or_ipsi	0.0099	0.0037	0.0082	0.0016	0.0039	0.0011	0.0063	0.0012
CA2_py_contra	0.0214	0.0018	0.0160	0.0025	0.0179	0.0027	0.0082	0.0011
CA2_or_contra	0.0008	0.0008	0.0003	0.0003	0.0003	0.0001	0.0002	0.0002
CA3_py_ipsi	0.3925	0.0264	0.3886	0.0225	0.4384	0.0063	0.5040	0.0136
CA3_or_ipsi	0.0143	0.0037	0.0134	0.0040	0.0132	0.0021	0.0186	0.0047
CA3_py_contra	0.1229	0.0128	0.1128	0.0141	0.2019	0.0129	0.1280	0.0084
CA3_or_contra	0.0030	0.0010	0.0022	0.0009	0.0038	0.0010	0.0026	0.0009
MS-DB	0.0867	0.0071	0.1010	0.0092	0.0665	0.0041	0.0659	0.0072
SUB	0.0706	0.0114	0.0605	0.0068	0.0566	0.0026	0.0340	0.0058
EC	0.0029	0.0009	0.0038	0.0015	0.0119	0.0030	0.0076	0.0023
MnR/PMnR	0.0018	0.0006	0.0007	0.0004	0.0027	0.0003	0.0011	0.0004
Nucleus Reuniens	0.0004	0.0003	0.0000	0.0000	0.0002	0.0001	0.0002	0.0002

	WT young	WT old	APP-KI young	APP-KI old
# of mice	7	10	10	8
# of starters	67±10	59±13	274±23	118±13
# of starter neurons/section	7±1	7±1	22±2	11±1
# of total labeled neurons	829±158	714±194	2829±339	1175±186
Overall connectivity	22.118±1.967	21.019±2.140	14.435±0.722	14.377±1.923

Figure Click here to access/download;Figure;Figures_NBD_round1_07062022-

Supplementary Table 2. CSI and PI Comparison Statistics

CSI Comparison	WT young vs APP-KI young		WT old vs APP-KI old	
	p value	significance	p value	significance
CA1_or_ipsi	0.000411	***	0.145710	n.s.
CA1_py_contra	0.303682	n.s.	0.026418	*
CA1_or_contra	0.324404	n.s.	0.877462	n.s.
CA2_py_ipsi	0.000411	***	0.083139	n.s.
CA2_or_ipsi	0.013575	*	0.359934	n.s.
CA2_py_contra	0.200689	n.s.	0.011655	*
CA2_or_contra	0.287330	n.s.	0.852941	n.s.
CA3_py_ipsi	0.600884	n.s.	0.896750	n.s.
CA3_or_ipsi	0.618624	n.s.	0.677179	n.s.
CA3_py_contra	0.108803	n.s.	0.965401	n.s.
CA3_or_contra	0.981798	n.s.	>0.999999	n.s.
MS-DB	0.000103	***	0.015540	*
SUB	0.314788	n.s.	0.054527	n.s.
EC	0.144655	n.s.	0.945221	n.s.
MnR/PMnR	0.193233	n.s.	0.243795	n.s.
Nucleus Reuniens	0.643665	n.s.	0.444444	n.s.

Statistical method: Wilcoxon rank sum test.
* p<0.05, ** p<0.01, *** p<0.001, **** p<0.0001

CSI Comparison	WT young vs WT old		APP-KI young vs APP-KI old	
	p value	significance	p value	significance
CA1_or_ipsi	0.600884	n.s.	0.011655	*
CA1_py_contra	0.023396	*	0.000046	****
CA1_or_contra	0.205728	n.s.	0.913913	n.s
CA2_py_ipsi	0.229535	n.s.	0.121989	n.s.
CA2_or_ipsi	0.886775	n.s.	0.203071	n.s.
CA2_py_contra	0.314788	n.s.	0.015540	*
CA2_or_contra	>0.999999	n.s.	0.230769	n.s.
CA3_py_ipsi	0.886775	n.s.	0.761826	n.s.
CA3_or_ipsi	>0.999999	n.s.	0.459756	n.s.
CA3_py_contra	0.812526	n.s.	0.043421	*
CA3_or_contra	0.679864	n.s.	0.713378	n.s.
MS-DB	0.600884	n.s.	0.514786	n.s.
SUB	0.600884	n.s.	0.274281	n.s.
EC	0.309524	n.s.	0.680594	n.s.
MnR/PMnR	0.060469	n.s.	0.100416	n.s.
Nucleus Reuniens	0.154412	n.s.	0.122172	n.s.

Statistical method: Wilcoxon rank sum test.
* p<0.05, ** p<0.01, *** p<0.001, **** p<0.0001

Figure

Click here to access/download;Figure;Figures_NBD_round1_07062022-

PI Comparison	WT young vs APP-KI young		WT old vs APP-KI old	
	p value	significance	p value	significance
CA1_or_ipsi	0.004628	**	0.315417	n.s.
CA1_py_contra	0.812526	n.s.	0.033937	*
CA1_or_contra	0.667781	n.s.	0.51017	n.s.
CA2_py_ipsi	0.000411	***	0.083139	n.s.
CA2_or_ipsi	0.018511	*	0.459756	n.s.
CA2_py_contra	0.474702	n.s.	0.026647	*
CA2_or_contra	0.295814	n.s.	0.852941	n.s.
CA3_py_ipsi	0.161251	n.s.	0.001371	**
CA3_or_ipsi	0.669066	n.s.	0.498697	n.s.
CA3_py_contra	0.001954	**	0.359934	n.s.
CA3_or_contra	0.739099	n.s.	0.492618	n.s.
MS-DB	0.02499	*	0.011655	*
SUB	0.474702	n.s.	0.01554	*
EC	0.072707	n.s.	0.119795	n.s.
MnR/PMnR	0.087824	n.s.	0.372275	n.s.
Nucleus Reuniens	0.643665	n.s.	0.444444	n.s.

Statistical method: Wilcoxon rank sum test.
* p<0.05, ** p<0.01, *** p<0.001, **** p<0.0001

PI Comparison	WT young vs WT old		APP-KI young vs APP-KI old	
	p value	significance	p value	significance
CA1_or_ipsi	0.417318	n.s.	0.002057	**
CA1_py_contra	0.013575	*	0.000046	****
CA1_or_contra	0.159348	n.s.	0.965355	n.s.
CA2_py_ipsi	0.108803	n.s.	0.003062	**
CA2_or_ipsi	0.962258	n.s.	0.121989	n.s.
CA2_py_contra	0.108803	n.s.	0.006216	**
CA2_or_contra	>0.999999	n.s.	0.219457	n.s.
CA3_py_ipsi	0.962258	n.s.	0.00032	***
CA3_or_ipsi	0.8116	n.s.	0.572604	n.s.
CA3_py_contra	0.474702	n.s.	0.003062	**
CA3_or_contra	0.448529	n.s.	0.513483	n.s.
MS-DB	0.417318	n.s.	0.696467	n.s.
SUB	0.536199	n.s.	0.003062	**
EC	0.750926	n.s.	0.32634	n.s.
MnR/PMnR	0.095023	n.s.	0.008478	**
Nucleus Reuniens	0.154412	n.s.	0.122172	n.s.

Statistical method: Wilcoxon rank sum test.
* p<0.05, ** p<0.01, *** p<0.001, **** p<0.0001

Figure

Click here to access/download;Figure;Figures_NBD_round1_07062022-

Supplementary Table 3.
Data Summary of Sex-specific CSI and PI Values

CSI Summary	WT young male		WT young female		WT old male		WT old female		APP-KI young male		APP-KI young female		APP-KI old male		APP-KI old female	
	Mean	SEM	Mean	SEM	Mean	SEM	Mean	SEM	Mean	SEM	Mean	SEM	Mean	SEM	Mean	SEM
CA1_or_ipsi	1.0983	0.1723	1.4178	0.3831	1.4881	0.2591	1.6518	0.5479	0.6510	0.0761	0.6008	0.0381	1.1744	0.2161	0.7798	0.1021
CA1_py_contra	0.4543	0.2352	0.5538	0.1561	0.1636	0.0630	0.2815	0.1664	0.2956	0.0629	0.4446	0.0514	0.0316	0.0169	0.0297	0.0213
CA1_or_contra	0.0083	0.0044	0.0265	0.0076	0.0099	0.0047	0.0079	0.0049	0.0122	0.0039	0.0107	0.0033	0.0137	0.0071	0.0083	0.0052
CA2_py_ipsi	1.3560	0.1026	1.3093	0.1301	1.8017	0.4379	1.8148	0.4275	0.7368	0.0876	0.7898	0.0966	1.2241	0.2039	0.8223	0.0812
CA2_or_ipsi	0.1423	0.0542	0.0805	0.0094	0.1098	0.0258	0.1034	0.0636	0.0448	0.0090	0.0296	0.0112	0.0750	0.0248	0.0409	0.0107
CA2_py_contra	0.2810	0.0534	0.2260	0.0289	0.1909	0.0587	0.2290	0.0556	0.1934	0.0601	0.2016	0.0594	0.0884	0.0180	0.0566	0.0089
CA2_or_contra	0.0140	0.0140	0.0000	0.0000	0.0025	0.0025	0.0017	0.0017	0.0024	0.0017	0.0038	0.0018	0.0022	0.0022	0.0000	0.0000
CA3_py_ipsi	4.6247	1.2095	4.9975	0.5595	4.8688	0.9141	4.4133	0.6264	4.3574	0.5123	4.4486	0.5778	5.7404	1.1893	3.9896	0.6296
CA3_or_ipsi	0.1913	0.0273	0.1560	0.0324	0.1934	0.0559	0.1523	0.1270	0.1372	0.0434	0.1286	0.0232	0.1793	0.0649	0.2092	0.0813
CA3_py_contra	1.4967	0.4797	1.5385	0.1361	1.3514	0.3278	1.4813	0.4555	2.0292	0.3612	2.1764	0.4054	1.5091	0.2532	0.9157	0.2229
CA3_or_contra	0.0403	0.0228	0.0280	0.0119	0.0380	0.0153	0.0167	0.0145	0.0293	0.0151	0.0510	0.0215	0.0222	0.0075	0.0319	0.0165
MS-DB	0.9353	0.0713	1.0673	0.1868	1.1152	0.1923	1.3778	0.3696	0.6346	0.0199	0.6462	0.0458	0.7089	0.1187	0.5196	0.1303
SUB	0.5720	0.1729	1.0805	0.2672	0.6632	0.0735	0.6825	0.0370	0.5360	0.0388	0.5746	0.0643	0.5332	0.1216	0.1243	0.0099
EC	0.0450	0.0257	0.0256	0.0047	0.0495	0.0230	0.0548	0.0474	0.1024	0.0626	0.1530	0.0374	0.1453	0.0534	0.0202	0.0114
MnR/PMnR	0.0256	0.0081	0.0136	0.0071	0.0054	0.0054	0.0129	0.0094	0.0200	0.0044	0.0333	0.0040	0.0227	0.0084	0.0024	0.0024
Nucleus Reuniens	0.0000	0.0000	0.0077	0.0062	0.0000	0.0000	0.0000	0.0000	0.0023	0.0011	0.0022	0.0009	0.0017	0.0017	0.0000	0.0000

PI Summary	WT young male		WT young female		WT old male		WT old female		APP-KI young male		APP-KI young female		APP-KI old male		APP-KI old female	
	Mean	SEM	Mean	SEM	Mean	SEM	Mean	SEM	Mean	SEM	Mean	SEM	Mean	SEM	Mean	SEM
CA1_or_ipsi	0.1071	0.0341	0.1162	0.0225	0.1303	0.0197	0.1301	0.0297	0.0735	0.0188	0.0604	0.0044	0.1053	0.0097	0.1086	0.0265
CA1_py_contra	0.0355	0.0152	0.0476	0.0152	0.0122	0.0047	0.0211	0.0106	0.0295	0.0034	0.0452	0.0064	0.0038	0.0020	0.0041	0.0027
CA1_or_contra	0.0006	0.0003	0.0022	0.0009	0.0007	0.0004	0.0006	0.0004	0.0011	0.0004	0.0010	0.0003	0.0012	0.0007	0.0013	0.0009
CA2_py_ipsi	0.1303	0.0329	0.1062	0.0020	0.1436	0.0129	0.1490	0.0261	0.0760	0.0046	0.0791	0.0087	0.1112	0.0093	0.1122	0.0194
CA2_or_ipsi	0.0152	0.0083	0.0060	0.0009	0.0087	0.0011	0.0076	0.0039	0.0052	0.0019	0.0026	0.0008	0.0067	0.0016	0.0057	0.0019
CA2_py_contra	0.0249	0.0019	0.0187	0.0021	0.0139	0.0027	0.0191	0.0048	0.0178	0.0046	0.0180	0.0034	0.0083	0.0015	0.0079	0.0021
CA2_or_contra	0.0018	0.0018	0.0000	0.0000	0.0005	0.0005	0.0001	0.0001	0.0002	0.0001	0.0003	0.0001	0.0002	0.0002	0.0000	0.0000
CA3_py_ipsi	0.3947	0.0600	0.3909	0.0191	0.4039	0.0291	0.3658	0.0374	0.4447	0.0057	0.4321	0.0112	0.4924	0.0145	0.5234	0.0270
CA3_or_ipsi	0.0185	0.0048	0.0112	0.0035	0.0152	0.0043	0.0107	0.0082	0.0132	0.0034	0.0132	0.0030	0.0142	0.0049	0.0259	0.0088
CA3_py_contra	0.1245	0.0256	0.1217	0.0181	0.1065	0.0153	0.1224	0.0293	0.1996	0.0210	0.2041	0.0174	0.1340	0.0061	0.1180	0.0214
CA3_or_contra	0.0040	0.0020	0.0023	0.0013	0.0030	0.0013	0.0011	0.0009	0.0026	0.0012	0.0049	0.0016	0.0019	0.0008	0.0038	0.0019
MS-DB	0.0878	0.0171	0.0860	0.0052	0.0955	0.0105	0.1091	0.0179	0.0683	0.0076	0.0646	0.0038	0.0636	0.0075	0.0698	0.0169
SUB	0.0489	0.0077	0.0869	0.0197	0.0619	0.0106	0.0585	0.0081	0.0562	0.0035	0.0570	0.0043	0.0444	0.0048	0.0166	0.0007
EC	0.0036	0.0022	0.0024	0.0007	0.0037	0.0017	0.0039	0.0032	0.0098	0.0057	0.0139	0.0023	0.0107	0.0027	0.0024	0.0013
MnR/PMnR	0.0027	0.0013	0.0011	0.0006	0.0005	0.0005	0.0010	0.0006	0.0020	0.0004	0.0033	0.0002	0.0016	0.0005	0.0003	0.0003
Nucleus Reuniens	0.0000	0.0000	0.0007	0.0007	0.0000	0.0000	0.0000	0.0000	0.0002	0.0001	0.0002	0.0001	0.0003	0.0003	0.0000	0.0000

	WT young male	WT young female	WT old male	WT old female	APP-KI young male	APP-KI young female	APP-KI old male	APP-KI old female
# of mice	3	4	6	4	5	5	5	3
# of starters	69±14	63±22	45±10	80±28	280±47	260±12	125±16	107±27
# of starter neurons/section	7±2	7±1	5±1	9±3	22±3	22±1	13±1	9±1
# of total labeled neurons	768±301	875±131	533±152	987±427	2974±611	2685±365	1373±227	846±257
Overall connectivity	20.657±3.108	23.214±2.789	20.487±3.156	21.818±3.015	13.791±0.982	15.078±1.08	16.513±2.677	10.817±0.685

Grey color filled areas represent male groups.

Figure

Click here to access/download;Figure;Figures_NBD_round1_07062022-

Supplementary Table 4.
Statistical Comparisons of WT and APP-KI Mouse Data Between Sexes

CSI Comparison	WT young male vs WT young female		WT old male vs WT old female		APP-KI young male vs APP-KI young female		APP-KI old male vs APP-KI old female	
	p value	significance	p value	significance	p value	significance	p value	significance
CA1_or_ipsi	0.628571	n.s.	>0.999999	n.s.	0.84127	n.s	0.392857	n.s
CA1_py_contra	0.942857	n.s.	0.609524	n.s.	0.134921	n.s	0.875	n.s
CA1_or_contra	0.114286	n.s.	0.714286	n.s.	0.587302	n.s	0.785714	n.s
CA2_py_ipsi	0.857143	n.s.	0.914286	n.s.	0.84127	n.s.	0.392857	n.s.
CA2_or_ipsi	0.228571	n.s.	0.609524	n.s.	0.309524	n.s.	0.571429	n.s.
CA2_py_contra	0.628571	n.s.	0.609524	n.s.	0.84127	n.s.	0.571429	n.s.
CA2_or_contra	0.428571	n.s.	>0.999999	n.s.	0.761905	n.s.	>0.999999	n.s.
CA3_py_ipsi	>0.999999	n.s.	0.914286	n.s.	0.84127	n.s.	0.392857	n.s.
CA3_or_ipsi	0.40000	n.s.	0.438095	n.s.	0.84127	n.s.	0.571429	n.s.
CA3_py_contra	>0.999999	n.s.	>0.999999	n.s.	0.690476	n.s.	0.142857	n.s.
CA3_or_contra	0.714286	n.s.	0.552381	n.s.	0.547619	n.s.	0.446429	n.s.
MS-DB	0.857143	n.s.	0.609524	n.s.	>0.999999	n.s.	0.571429	n.s.
SUB	0.114286	n.s.	0.761905	n.s.	>0.999999	n.s.	0.035714	*
EC	0.628571	n.s.	>0.999999	n.s.	0.309524	n.s.	0.142857	n.s.
MnR/PMnR	0.628571	n.s.	0.4	n.s.	0.055556	n.s.	0.142857	n.s.
Nucleus Reuniens	0.428571	n.s.	>0.999999	n.s.	0.825397	n.s.	>0.999999	n.s.

Statistical method: Wilcoxon rank sum test.
* p<0.05, ** p<0.01, *** p<0.001, **** p<0.0001

PI Comparison	WT young male vs WT young female		WT old male vs WT old female		APP-KI young male vs APP-KI young female		APP-KI old male vs APP-KI old female	
	p value	significance	p value	significance	p value	significance	p value	significance
CA1_or_ipsi	0.857143	n.s.	0.914286	n.s.	>0.999999	n.s.	>0.999999	n.s.
CA1_py_contra	0.628571	n.s.	0.476190	n.s.	0.095238	n.s.	>0.999999	n.s.
CA1_or_contra	0.057143	n.s.	0.904762	n.s.	0.460317	n.s.	>0.999999	n.s.
CA2_py_ipsi	0.857143	n.s.	0.914286	n.s.	0.690476	n.s.	>0.999999	n.s.
CA2_or_ipsi	0.400000	n.s.	0.257143	n.s.	0.095238	n.s.	0.571429	n.s.
CA2_py_contra	0.114286	n.s.	0.476190	n.s.	0.841270	n.s.	>0.999999	n.s.
CA2_or_contra	0.428571	n.s.	>0.999999	n.s.	0.841270	n.s.	>0.999999	n.s.
CA3_py_ipsi	0.857143	n.s.	0.476190	n.s.	0.420635	n.s.	0.392857	n.s.
CA3_or_ipsi	0.400000	n.s.	0.438095	n.s.	>0.999999	n.s.	0.250000	n.s.
CA3_py_contra	>0.999999	n.s.	0.761905	n.s.	>0.999999	n.s.	0.785714	n.s.
CA3_or_contra	0.457143	n.s.	0.438095	n.s.	0.309524	n.s.	0.446429	n.s.
MS-DB	0.857143	n.s.	0.761905	n.s.	>0.999999	n.s.	>0.999999	n.s.
SUB	0.114286	n.s.	>0.999999	n.s.	0.841270	n.s.	0.035714	*
EC	0.857143	n.s.	>0.999999	n.s.	0.690476	n.s.	0.071429	n.s.
MnR/PMnR	0.400000	n.s.	0.500000	n.s.	0.031746	*	0.142857	n.s.
Nucleus Reuniens	0.428571	n.s.	>0.999999	n.s.	>0.999999	n.s.	>0.999999	n.s.

Statistical method: Wilcoxon rank sum test.
* p<0.05, ** p<0.01, *** p<0.001, **** p<0.0001
